# Persistent EGFR/K-RAS/SIAH pathway activation drives chemo-resistance and early tumor relapse in triple-negative breast cancer

**DOI:** 10.20517/cdr.2022.31

**Published:** 2022-06-22

**Authors:** Amy H. Tang, Richard A. Hoefer, Mary L. Guye, Harry D. Bear

**Affiliations:** ^1^Department of Microbiology and Molecular Cell Biology, Leroy T. Canoles Jr. Cancer Research Center, Eastern Virginia Medical School, Norfolk, VA 23507, USA.; ^2^School of Systems Biology, George Mason University, Manassas, VA 20110, USA.; ^3^Sentara Cancer Network, Sentara Healthcare, Norfolk, VA 23507, USA.; ^4^Sentara Surgery Specialists, Surgical Oncology, Sentara CarePlex Hospital, Newport News, VA 23606, USA.; ^5^Division of Surgical Oncology, Departments of Surgery and Microbiology and Immunology, Massey Cancer Center, Virginia Commonwealth University, Richmond, VA 23298-0011, USA.

**Keywords:** Triple-negative breast cancer (TNBC), chemo-resistance, seven in absentia (SINA) and human homologs of SINA (SIAH) E3 ligase, ubiquitin-mediated proteolysis, EGFR/K-RAS/SIAH pathway activation in TNBC, neoadjuvant chemotherapy prognosis, patient risk stratification, detection of chemo-resistance, precision quantification of therapy efficacy, and treatment optimization

## Abstract

Triple-negative breast cancer (TNBC) is the most aggressive breast cancer subtype. It disproportionately affects BRCA mutation carriers and young women, especially African American (AA) women. Chemoresistant TNBC is a heterogeneous and molecularly unstable disease that challenges our ability to apply personalized therapies. With the approval of immune checkpoint blockade (ICB) for TNBC, the addition of pembrolizumab to systemic chemotherapy has become standard of care (SOC) in neoadjuvant systemic therapy (NST) for high-risk early-stage TNBC. Pembrolizumab plus chemotherapy significantly increased the pathologic complete response (pCR) and improved event-free survival in TNBC. However, clinical uncertainties remain because similarly treated TNBC partial responders with comparable tumor responses to neoadjuvant therapy often experience disparate clinical outcomes. Current methods fall short in accurately predicting which high-risk patients will develop chemo-resistance and tumor relapse. Therefore, novel treatment strategies and innovative new research initiatives are needed. We propose that the EGFR-K-RAS-SIAH pathway activation is a major tumor driver in chemoresistant TNBC. Persistent high expression of SIAH in residual tumors following NACT/NST reflects that the EGFR/K-RAS pathway remains activated (ON), indicating an ineffective response to treatment. These chemoresistant tumor clones persist in expressing SIAH (SIAH^High/ON^) and are linked to early tumor relapse and poorer prognosis. Conversely, the loss of SIAH expression (SIAH^Low/OFF^) in residual tumors post-NACT/NST reflects EGFR/K-RAS pathway inactivation (OFF), indicating effective therapy and chemo-sensitive tumor cells. SIAH^Low/OFF^ signal is linked to tumor remission and better prognosis post-NACT/NST. Therefore, SIAH is well-positioned to become a novel tumor-specific, therapy-responsive, and prognostic biomarker. Potentially, this new biomarker (SIAH^High/ON^) could be used to quantify therapy response, predict chemo-resistance, and identify those patients at the highest risk for tumor relapse and poor survival in TNBC.

## INTRODUCTION

### Triple-negative breast cancer

Triple-negative breast cancer (TNBC) represents 15%-20% of all breast cancers diagnosed in the United States, and it is characterized by the absence of estrogen receptors (ER), progesterone receptors (PR), and human epidermal growth factor receptor 2 (HER2)^[1-5]^. https://seer.cancer.gov/statfacts/html/breast.html. TNBC is the most aggressive subtype of breast cancer, and it disproportionately affects *BRCA1* mutation carriers and young women, especially those with Western African ancestry^[6-13]^. This molecular subtype is nearly twice as common in African American women (AA) than in Caucasian women^[6,8,9,14-17]^. TNBC is a genetically diverse, highly heterogeneous, and molecularly unstable disease, which challenges our ability to tailor effective individualized treatments for patients^[10,18]^. TNBC has unique and aggressive tumor biology, and it constitutes a major health threat with the worst prognosis and the highest mortality of all breast cancer subtypes^[10,19,20]^. Looking more closely, one in three patients with high-risk early-stage TNBC will develop tumor relapse, which typically occurs within the first three years of initial diagnosis; only a third of women with locoregional TNBC will survive their disease; and of those with metastatic TNBC, less than 1 in 9 will survive their disease^[5,10,21-23]^. TNBC has a 5-year overall survival (OS) of 78.5%, and the 5-year survival rates for localized, regional, and metastatic diseases are 91.2%, 65.4%, and 12.2%, respectively, which is the worst among the major molecular subtypes in breast cancer (https://seer.cancer.gov/explorer/application.html?site=623&data_type=4&graph_type=5&compareBy=stage&chk_stage_101=101&chk_stage_104=104&chk_stage_105=105&chk_stage_106=106&series=9&hdn_sex=3&race=1&age_range=1&advopt_precision=1&advopt_show_ci=on&advopt_display=2). Thus, the dismal prognosis, chemo-resistance, and high mortality of regional and metastatic TNBC highlights a critical unmet need for the development of improved therapies and the discovery of reliable prognostic biomarkers such as SIAH, which can single out the highest risk patients at the first-line neoadjuvant setting, identify chemoresistant tumors that are difficult to treat and prone to develop early relapse, optimize effective treatment sequences, and select the best combinational strategies for better clinical outcomes and prolonged survival.

### Standard treatment regimens in TNBC

Standard chemotherapy remains the backbone of systemic therapy in TNBC^[20,24-28]^. https://www.cancer.org/cancer/breast-cancer/treatment/treatment-of-triple-negative.html. Neoadjuvant chemotherapy (NACT) was the previous standard of care (SOC) to treat high-risk and locally advanced TNBC prior to July 26, 2021^[10,29]^. The addition of immune checkpoint blockade (ICB) to chemotherapy is now the current SOC for neoadjuvant systemic therapy (NST) to treat high-risk early-stage TNBC. Immuno-oncology (IO) therapy is an exciting scientific breakthrough in the treatment of TNBC^[30-32]^. Immunotherapy that targets programmed death receptor-1 (PD-1) has shown great promise in treating a subset of TNBC patients in combination with chemotherapy^[33,34]^. Pembrolizumab plus chemotherapy significantly improved the pCR rates in high-risk early-stage TNBC in the neoadjuvant setting^[35,36]^. As shown in the KEYNOTE-522 trial, neoadjuvant pembrolizumab plus chemotherapy led to an improved pCR rate (65%) in high-risk early-stage TNBC^[35]^. Notably, the TNBC pIR patients with residual disease at the time of surgery seemed to benefit the most from the addition of IO-therapy in the neoadjuvant and adjuvant settings^[37]^. Surprisingly, the 3-year event-free survival (EFS) benefit associated with pembrolizumab was independent of PD-L1 expression in high-risk early-stage TNBC^[35]^. Based on the I-SPY 2 trial, the pCR rate doubled to 60% when pembrolizumab was added to standard chemotherapy to treat stage II/III TNBC patients with T2/N1 or higher stage tumors^[30,36]^. In contrast, as reported in KEYNOTE-355, KEYNOTE-119, Impassion130, and Impassion131, PD-(L)1-targeted immunotherapies plus chemotherapy have shown only modest survival benefit in PD-L1-positive TNBC in advanced and metastatic settings^[33,34,38]^. At the same time, unfortunately, the grade 3 or 4 treatment-related adverse events were significantly increased in response to the new IO-regimens^[39]^.

### Unmet needs in TNBC

As more and more TNBC patients are treated with pembrolizumab plus chemotherapy in both neoadjuvant and/or metastatic settings, ICB resistance is evidently emerging, and serious adverse side effects were reported in a subset of TNBC patients^[35,38]^. Despite the benefit of this newly FDA-approved IO-therapy, 30%-44% of high-risk early-stage TNBC patients and 60%-70% of PD-L1-positive metastatic TNBC patients who receive the IO-therapy did not show any objective improvement^[35,38]^. Without a proper guide, pembrolizumab plus chemotherapy is often administered “blindly” in the neoadjuvant setting following the newly FDA-approved standard IO-regimens to treat high-risk early-stage TNBC. How to maximize the current SOC chemo- and IO-therapy in combination while limiting chemo- and IO-resistance, and minimizing the side effects of immunotherapy is a difficult problem and an unmet need for a large number of TNBC patients.

Pathology following completion of NACT/NST, with or without immunotherapy (pembrolizumab), produces a binary response: pathologic complete response (pCR) or pathologic incomplete response (pIR)^[40,41]^. pCR is a reliable prognostic marker that correlates with tumor remission and long-term survival, whereas pIR is associated with an increased risk of early tumor relapse and poor prognosis^[36,42-47]^. Incomplete responders can be further classified by the residual cancer burden (RCB classes I-III); the higher the RCB classification, the higher the likelihood of tumor relapse and mortality^[21,40,41,48-51]^. TNBC patients with high-risk and high-grade residual disease are now commonly treated with additional adjuvant chemotherapy, including capecitabine, which may be combined with immunotherapy (pembrolizumab) post-operatively^[35,36,38,52-54]^. Clinical uncertainties remain, because although many TNBC patients with the identical clinical and pathological tumor stages by the American Joint Committee on Cancer (AJCC) TNM classification, and similar residual cancer burden (RCB) after a non-pCR (pIR) diagnosis post-NACT/NST, will often experience disparate clinical outcomes and survival^[55,56]^. Current methods to stratify these high-risk patients fall short in predicting the risk of tumor recurrence and forecasting survival. There is no reliable prognostic biomarker that can be used to predict with certainty and molecular precision which RCB (I-II-III) tumors will stay in remission and which ones will relapse rapidly^[3]^. Few therapeutic agents, alone or in combination, are effective at eradicating chemoresistant and metastatic TNBC^[57-60]^. Therefore, the development of a new tumor-specific biomarker that can be used to stratify high-risk TNBC patients in the first-line neoadjuvant setting, quantifying treatment efficacy in real time in the clinical setting is essential. Additionally, utilizing this same biomarker to detect the emergence of chemoresistant tumor clones at a single tumor cell resolution, forecast risk for early tumor relapse, and predict patient survival would equip us with a new therapy-responsive prognostic biomarker to quantify, guide, and treat TNBC more effectively^[3]^.

### Chemo-resistance in TNBC

Chemo-resistance is a vexing problem and a major life-threatening feature of TNBC^[25,57,61]^. Activation of multiple signaling pathways, context-dependent compensatory pathway cross-talk, synergy, antagonism, and signaling network “rewiring” are all implicated in the development of chemoresistant phenotypes in TNBC. These include Wnt/β-catenin, Notch, Hedgehog, NFκB, PI3K/mTOR, Hippo/YAP, JAK/Stat, TGFβ, hypoxia, p53 loss of function and BRCA mutations, altered metabolism, and increased transporter and efflux pump activity^[25,57,62-66]^. Single-cell sequencing has revealed that plasticity, heterogeneity, rapid molecular evolution of innate and acquired chemo-resistance, cellular senescence, and dynamic remodeling of epithelial-mesenchymal transition (TME)/mesenchymal-epithelial transition (MET) states of tumor-initiating cells or cancer stem cells in TNBC contribute to cancer recurrences^[49,62,67-71]^. These aforementioned topics have been reviewed extensively in the TNBC literature. Here, our discussion will focus on persistent activation of the EGFR/K-RAS/MAPK/SIAH pathway, which drives chemo-resistance, early tumor relapse, and high mortality in TNBC^[3,72]^.

### Persistent EGFR/K-RAS/MAPK/SIAH pathway activation drives TNBC malignancy

Genomic landscape studies indicate that EGFR/K-RAS/MAPK pathway activation is a major impetus driving TNBC malignancy, early tumor relapse, local invasion, and metastatic spread^[73,74]^. Aberrant EGFR/K-RAS/MAPK/SIAH pathway activation is highly prevalent in chemoresistant, recurrent, locally advanced, and metastatic TNBC^[3,75-78]^. Heightened EGFR/K-RAS/MAPK activation has multiple deleterious effects on the tumor/tumor microenvironment (TME), which is associated with decreased tumor-infiltrating lymphocytes (TIL) detection in TNBC and is correlated with increased metastases and poor prognosis in breast cancer^[73,74,78]^. With the new FDA-approved chemo- and immunotherapy combination to treat high-risk early-stage TNBC, it is important to maximize the therapeutic benefit and identify chemoresistant tumor cells as early as possible, but also minimize the adverse toxicities and immune side-effects of these IO-combination therapies in the neoadjuvant and adjuvant settings.

### SIAH is the most conserved downstream signaling gatekeeper in the EGFR/K-RAS/MAPK signaling pathway

**Figure 1:**
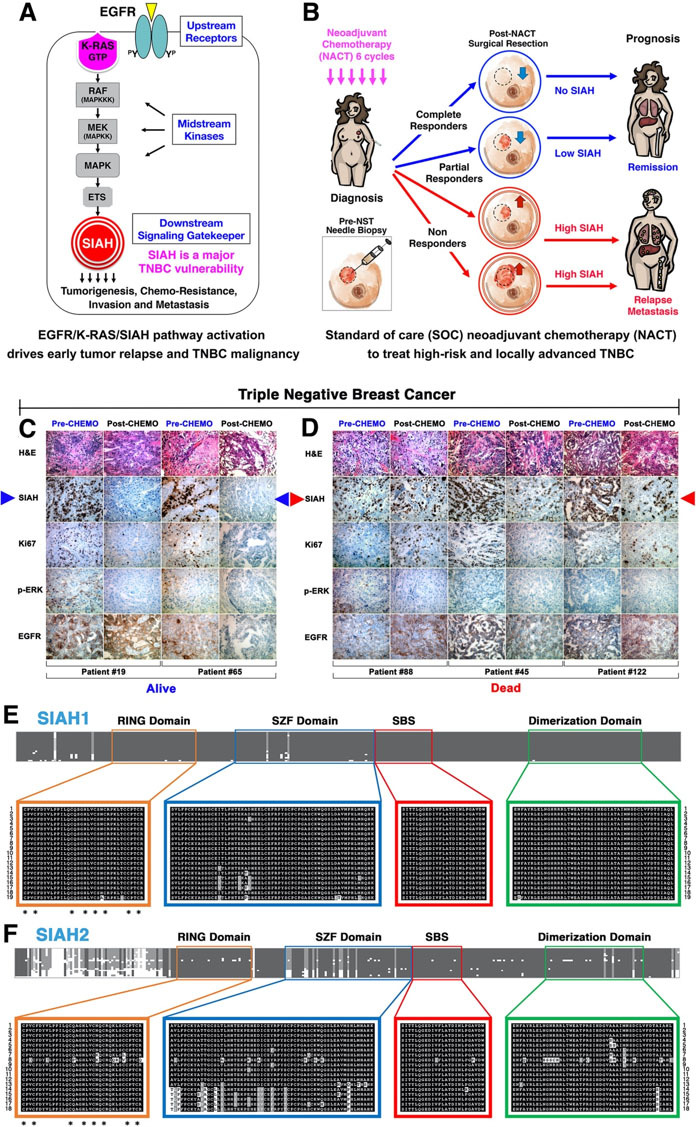
SIAH is the most conserved downstream signaling gatekeeper in the EGFR/K-RAS/SIAH pathway, whose persistent activation is driving TNBC malignancy, tumor relapse, and metastasis. (A) SIAH is the most evolutionarily conserved and the most downstream signaling module identified in the EGFR/K-RAS signaling pathway thus far. (B) Loss of SIAH expression (SIAH^Low/OFF^) after effective NACT is correlated with EGFR/K-RAS pathway inactivation and tumor regression/remission, whereas persistent SIAH expression (SIAH^High/ON^) after ineffective NACT is correlated with EGFR/K-RAS pathway activation and tumor progression/early relapse. Persistent high SIAH expression (SIAH^High/ON^) in high-risk residual tumors post NACT is correlated with EGFR/K-RAS pathway activation, chemo-resistance, and early tumor relapse. (C-D) TNBC tumors were stained with H&E, SIAH, Ki67, phospho-ERK, and EGFR. SIAH outperforms Ki67. SIAH is prognostic and Ki67 is not prognostic in NACT-treated high-risk and locally advanced breast cancer. We found that SIAH^Low/OFF^ post-NACT correlates with tumor remission and prolonged survival (Alive at 5 years) (C). We found that persistent SIAH^High/ON^ expression in residual tumors post-NACT is associated with tumor relapse and poor survival (Dead before3-5 years) (D). (E) SIAH1 and (F) SIAH2 are extraordinarily conserved across metazoan species. Conclusion: We found that SIAH^ON/OFF^ expression is a binary code that reflects tumor-driving EGFR/K-RAS/SIAH pathway activation^ON^/inactivation^OFF^ in TNBC primary and residual tumors As such, SIAH is strategically well positioned to become a new TNBC target, and a new tumor-specific, therapy-responsive, and prognostic biomarker to risk-stratify pIR patients, detect the emergence of treatment-refractory tumors, quantify NACT/NST efficacy, augment RCB classifications, forecast early relapse, and predict patient survival in real time in the clinic. SIAH: Human homologs of *Drosophila* Seven In Absentia (SINA); NACT: neoadjuvant chemotherapy; RCB: residual cancer burden; TNBC: triple-negative breast cancer.

Due to the extraordinary conservation of EGFR/RAS/MAPK/SIAH signaling pathway across metazoan species, the molecular insights and core principles gleaned from *Drosophila* EGFR/RAS/SINA studies have shed light on the evolutionarily conserved principles and fundamental aspects of mammalian EGFR/K-RAS/MAPK/SIAH signaling pathway, and guided anti-EGFR/K-RAS/MAPK/SIAH drug development in human cancer^[77,79-85]^. As a RING-domain E3 ubiquitin ligase, the human homologs of SINA (SIAH) or *Drosophila* Seven-In-Absentia (SINA) are the most downstream gatekeeper and the most evolutionarily conserved signaling component in the EGFR/K-RAS/MEK/MAPK pathway identified thus far [Figure 1A, 1E and 1F]^[72,77,82-88]^. Due to its conserved signaling gatekeeper function as a major network bottleneck, SIAH^ON/OFF^ expression is well-positioned to serve as a direct readout of tumor-driving EGFR/K-RAS/MEK/MAPK pathway activation (ON)/inactivation (OFF) in TNBC [Figure 1B]^[76,77,85,89]^. SIAH^Low/OFF^ in TNBC post-NACT correlates with tumor remission, effective therapy, and good prognosis [Figure 1C], whereas SIAH^High/ON^ in TNBC post-NACT correlates with early relapse, ineffective therapy, and poor survival [Figure 1D]^[76]^. Therefore, SIAH is likely to be an excellent prognostic biomarker to stratify incomplete responders in the first-line neoadjuvant setting^[3,76,89]^. SIAH may be used to identify chemoresistant tumor cells as early as possible in the neoadjuvant setting as a therapy-responsive biomarker in order to identify the difficult-to-treat cancers at the highest-risk for early relapse and treatment-resistance. Furthermore, we propose that SIAH can be used to augment residual cancer burden (RCB I-III) classification in quantifying the efficacy of SOC treatment regimens, detect chemoresistant tumor clones, forecast early tumor relapse, and predict patient survival. Having this accuracy in real time allows the clinician to pivot with precision to select more effective drugs and optimize the sequence of combination therapies for treatment-refractory TNBC [Figure 1]^[3,76,77,85]^.

### The SIAH^ON/OFF^ binary code reflects this major tumor-driving EGFR/K-RAS/MAPK pathway activation (ON) and inactivation (OFF) in TNBC

Supported by strong evidence in developmental, evolutionary, and cancer biology, we hypothesize that persistent EGFR–K-RAS–SIAH pathway activation is a major tumor-driving force in TNBC, and that SIAH is a new tumor-specific, therapy-responsive, and prognostic biomarker for patient risk stratification, therapy quantification, and treatment optimization^[76,77,82-85,89]^. We propose that the persistent high expression of SIAH (SIAH^High/ON^) post-NACT/NST reflects tumor-driving EGFR/K-RAS/MAPK pathway activation (ON), resulting in tumor progression, immuno-suppression, and chemo-resistance, versus the loss of SIAH expression (SIAH^Low/OFF^) post-NACT/NST reflects this tumor-driving pathway is inactivated (OFF), resulting in tumor regression, immune responsiveness, and chemo-sensitivity^[3,76]^. This new tumor-specific, therapy-responsive, and prognostic SIAH^ON/OFF^ binary code can potentially be used to identify those TNBC pIR patients at the highest risk for early tumor relapse, detect multidrug-resistant residual tumor clones in real time, combine and guide precise therapies in the first-line setting.

### Clinical utility of SIAH as a tumor-specific, therapy-responsive, and prognostic biomarker for risk stratification, early relapse, and survival prediction in TNBC

**Figure 2:**
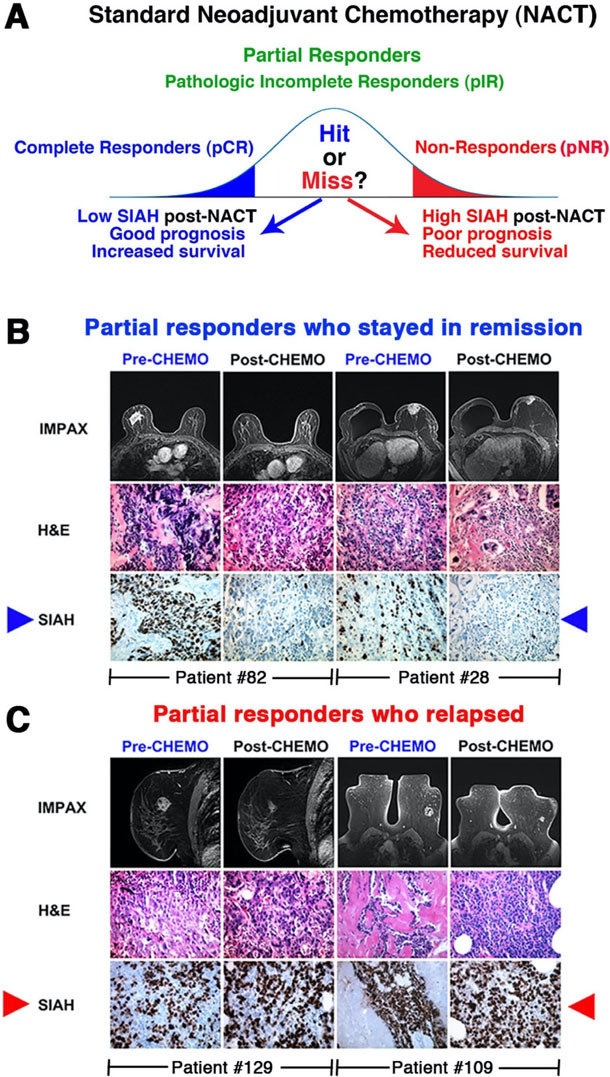
SIAH marks proliferating tumor cells at a single-cell resolution, and SIAH is a therapy-responsive and prognostic biomarker that can be used to risk-stratify incomplete responders in NACT/NST-treated high-risk and locally advanced breast cancer. IMPAX stands for the IMPAX digital mammography, a powerful diagnostic platform for breast imaging at the Sentara Breast Centers. (A) pCR is a good prognostic marker associated with long-term survival post-NACT/NST. However, for the pIR patients with residual disease, additional tools are needed to distinguish which patients are at high risk for early tumor relapse and thus who may need additional adjuvant chemotherapies. (B) The pIR patients with no or low SIAH expression in residual tumors post-NACT/NST stayed in remission and have prolonged survival (examples: patients #82 and #28). (C) In contrast, the pIR patients with persistent high SIAH expression in residual disease post-NACT/NST developed early relapse and succumbed to their chemoresistant and metastatic diseases (examples: patients #109 and #129). Conclusion: We propose that persistent high SIAH expression in residual tumors is associated with early tumor relapse and poor prognosis, while no or low SIAH expression in residual tumors is associated with tumor remission and good prognosis post-NACT/NST. SIAH: Human homologs of *Drosophila* Seven In Absentia (SINA); Pir: pathologic incomplete response; NACT: neoadjuvant chemotherapy; NST: neoadjuvant systemic therapy; PCR: athologic complete response.

We generated an anti-SIAH monoclonal antibody for tumor IHC/IF/FACS staining^[82,83]^. Our pilot study of 57 NACT-treated TNBC patients with residual disease showed that SIAH^Low/OFF^ correlates with tumor remission and good prognosis [Figure 1C]. For those with SIAH^High/ON^ in their residual tumors, it predicts chemoresistant tumor cells/clones, early tumor relapse, and poor prognosis post-NACT/NST [Figure 1D]^[76]^. The prognostic impact of SIAH expression seemed to be far superior to that of Ki67 and phospho-ERK in NACT/NST-treated breast cancer^[76]^. SIAH could be used to risk stratify incomplete responders, identify chemo- and IO-resistance, quantify therapy efficacy, and predict relapse and survival [Figure 2A]. SIAH^Low/OFF^ in TNBC residual tumors reflects that the EGFR/K-RAS/MAPK/SIAH pathway is OFF, and indicates chemo-sensitivity, effective NACT/NST, and good prognosis after surgery [Figure 2B]. SIAH^High/ON^ in TNBC residual tumors reflects that the EGFR/K-RAS/MAPK/SIAH pathway is ON, which indicates chemo-resistance, ineffective NACT/NST, and/or the need for additional adjuvant therapies to prevent progressive disease and early tumor relapse [Figure 2C]. Our studies have demonstrated that SIAH offers a tumor-specific, therapy-responsive, and prognostic biomarker in TNBC with high molecular precision and full dynamic range (0%-100%)^[76,89]^. Therefore, SIAH^ON/OFF^ expression can be used to identify chemoresistant tumor clones, differentiate partial responders, forecast tumor relapse, and predict patient survival in NACT-treated breast cancer^[76,89]^. Moreover, we have shown that SIAH^ON/OFF^ expression is a direct readout of EGFR/K-RAS/MAPK pathway activation (ON)/inactivation (OFF)^[3,76,77,84,85]^. Hence, studying the “ON/OFF” of the major tumor-driving EGFR/K-RAS/MAPK/SIAH pathway may represent an opportunity to risk-stratify TNBC patients before and after NACT/NST (IO-therapy). As a binary biomarker with high tumor specificity and detection sensitivity as well as a full dynamic range (0%-100%), we propose to validate that SIAH is a powerful new prognostic biomarker that can be used to risk-stratify patients, detection of chemo-resistance, quantify NACT/NST efficacy, forecast early tumor relapse, and predict patient survival in TNBC^[72,76,77,82,83]^.

## CONCLUSION

Focusing on EGFR/K-RAS/MAPK/SIAH pathway activation as a major tumor driver in TNBC to risk-stratify patients and detect chemo-resistance may represent a significant step forward. In our pilot study, we found that all TNBC primary tumors are highly proliferative, heavily decorated with SIAH, and the median SIAH expression in untreated TNBC tumors was 70% in both lymph node (LN) positive and LN-negative subsets^[76]^. In contrast, 30% of patients with residual disease displayed persistent high SIAH expression and had high relapse rates and poor outcomes^[76]^. As a tumor-specific, therapy-responsive, and prognostic biomarker in TNBC, SIAH has a full dynamic range, high sensitivity, high specificity, and molecular precision and could be used to risk-stratify patients and detect chemoresistant tumor cells in the first-line neoadjuvant settings. Ultimately, we hope to translate our findings (SIAH as a new prognostic biomarker) for clinical use to facilitate early detection of ineffective therapy in the first-line neoadjuvant setting, detect chemoresistant tumor cells at a single tumor cell resolution, augment RCB I-II-III classifications with high-precision, accurately calculate the risk of early tumor relapse and predict long-term survival in TNBC. If successful, we can use this new, dynamic, therapy-responsive, interactive, and tumor-specific biomarker, SIAH, to address the unmet need of identifying chemoresistant TNBC, and risk-stratify pIR patients by tumor relapse and poor prognosis in the first-line neoadjuvant setting. By leveraging the tumor-driving EGFR/K-RAS/SIAH pathway activation (ON)/inactivation (OFF) in TNBC, we hope to differentiate TNBC pIR patients by correlating SIAH^High^ (high-risk) versus SIAH^Low^ (low-risk) expression in residual tumors post-NACT/NST. This precision biomarker may also be used to detect treatment disparity amongst incomplete responders, forecast early tumor relapse, and predict survival. The next steps should include the successful executions of several independent large-scale multicenter biomarker validation studies leading to FDA approval of SIAH as a new tumor-specific, therapy-responsive, and prognostic biomarker for TNBC risk stratification, detection of chemo-resistance, therapy quantification in real time, treatment optimization in the clinic. Importantly, SIAH is a strategically positioned cancer target for us to develop a new anti-TNBC targeted therapy to eradicate multidrug-resistant, undruggable, and incurable TNBC malignancy in the future.

## DECLARATIONS

### Copyright

© The Author(s) 2022.
